# Plugging performance and mechanism of an OBDF oil-absorbing resin (MMA-SMA-St) plugging agent

**DOI:** 10.1039/d4ra02420f

**Published:** 2024-07-04

**Authors:** Yang Bai, Jianing He, Jinsheng Sun, Ren Wang, Ruifang Wang

**Affiliations:** a State Key Laboratory of Oil and Gas Reservoir Geology and Exploitation, School of Oil & Natural Gas Engineering, Southwest Petroleum University Chengdu 610500 China w18351551338@126.com; b College of Petroleum Engineering, Liaoning Petrochemical University Fushun 113001 China; c Key Laboratory of Unconventional Oil & Gas Development (China University of Petroleum (East China)), Ministry of Education Qingdao 266580 China

## Abstract

To treat the issue of increased resource wastage due to the higher plugging tendencies of oil-based drilling fluids (OBDF) relative to water-based drilling fluids, this study synthesized a ternary composite oil-absorbing resin and optimized its synthesis parameters. The influence of temperature variations on the resin's oil absorption capacity was assessed. Techniques such as infrared spectroscopy, scanning electron microscopy, TGA-DSC measurements, crosslinking degree analysis, contact angle analysis, X-ray photoelectron spectrometry analysis and examination of the resin's plugging mechanism were employed to investigate its molecular structure, oil absorption properties, and plugging efficiency. Additionally, the impact of various synthesis conditions on the oil absorption expansion rate of the oil-absorbing resin was examined. The findings indicate that the resin developed in this research maintains robust oil absorption capabilities at 160 °C, exhibiting an oil absorption expansion rate of 12.5 g g^−1^. At this temperature, the composite resin particles effectively sealed leaks of widths 0.25, 0.5, and 0.75 μm. Comparative analysis revealed that adding 3% of these resin particles to OBDF significantly enhanced the sealing of fractures. Remarkably, at 160 °C, OBDF amended with resin particles managed to completely seal fractures measuring 0.25 μm. The novelty of this study is attributed to the utilization of styrene for enhancing the resin's rigidity, coupled with the application of octadecyl methacrylate, which contains long-chain alkyl groups, to optimize the oil absorption and expansion characteristics of the oil-absorbing resin.

## Abstract 2

The innovation of this article lies in providing an oil absorbing resin plugging agent with good plugging effect.

## Graphical abstract

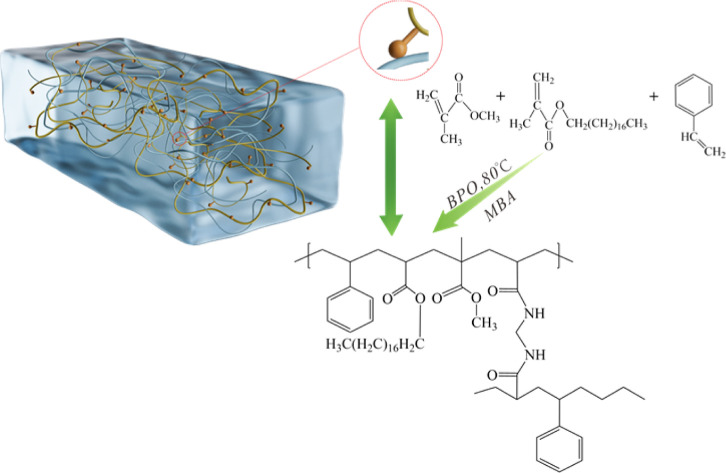

## Introduction

In recent years, the exploration and development of oil and gas wells have increasingly shifted toward unconventional, highly challenging, and complex wells, including deep and ultra-deepwater wells.^1–3^ Consequently, the demand for drilling fluids in the oil and gas development process has risen.^4^ Drilling fluid, a critical component of natural gas and oil extraction, serves to carry and suspend rock particles, stabilize wellbore walls, and balance formation pressure. It includes a variety of circulating fluids used across a broad spectrum of drilling operations in the oil and gas industry.^5,6^ During drilling, fragile and complex shale rock formations are susceptible to wellbore leakage, potentially resulting in issues such as sticking, blowouts, and thereby leading to significant economic losses and safety hazards, ultimately impairing drilling efficiency.^7^ Research shows that well leakage is responsible for 20% to 25% of all drilling projects, globally, leading to annual economic losses up to $9 billion in the petroleum industry worldwide.^8^ Addressing well leakage at drilling sites is, therefore, an imperative challenge in the drilling fluid field.

Currently, the majority of drilling fluids used in oil and gas field drilling engineering are categorized into water-based drilling fluid (WBDF),^9–11^ OBDF^12–15^ and synthetic-based drilling fluid.^16,17^ Among these, OBDF is often the preferred option for ultra-deep and complex wells due to its high-temperature resistance, superior lubrication, and enhanced wellbore stability. However, as OBDF systems continue to evolve, the issue of well leakage has become increasingly evident. On the one hand, the predominantly hydrophilic nature of the original rock surfaces within formations and the use of wetting agents in OBDF transform these surfaces from hydrophilic to oleophilic. Conversely, the requirement for oil-based drilling fluid plugging materials to exhibit excellent dispersibility in the oil phase necessitates that these materials possess an abundance of lipophilic functional groups. Consequently, this characteristic prevents the plugging material from forming non-covalent interactions, such as ionic and hydrogen bonds, with the oleophilic rock wall. During drilling, capillary forces can cause the wellbore wall rocks to adsorb both water and oil phases, enlarging the gap between fractured rock layers and thus heightening the risk of well leakage. Additionally, the effective lubrication provided by OBDF facilitates its entry into fractures, potentially inducing hydraulic fracturing and consequent well leakage.^18^ Consequently, the exploration of OBDF-compatible plugging materials has emerged as a prominent research topic. Currently, over a hundred types of plugging materials are utilized at major drilling sites, yet the vast majority are designed for WBDF, with only a limited number being compatible with OBDF.^19,20^ As a result, in the event of well leakage, technicians often resort to WBDF-appropriate plugging materials, which tend to yield suboptimal sealing outcomes. In summary, identifying a plugging material that exhibits strong compatibility with OBDF represents a significant area of interest within the field.

Currently, the plugging materials deemed compatible with OBDF predominantly encompass oleophilic resins, oil-absorbing and expanding substances, oil-based cross-linked gels, and hydrophobic materials.^21–23^ Among these, resin-based materials have attracted extensive research interest owing to their excellent compatibility with OBDF. For instance, Li *et al.*^14^ developed butadiene styrene/nano-SiO_2_ (SBR/SiO_2_) composites through a continuous emulsion polymerization process. Their findings revealed that the integration of silica and styrene not only imparts significant pressure resistance to the composites but also enables SBR/SiO_2_ to penetrate shale formations where leakage occurs, substantially mitigating fluid intrusion. Additionally, Du^30^ synthesized nanometer polystyrene dodecyl acrylate (PSL) oil absorption resin using styrene (St) and lauryl acrylate (LMA) *via* a soap-free emulsion polymerization method. This process yielded a product with remarkable dispersion stability and oil absorption expansion capabilities, primarily attributed to the flexible macromolecular chains of dodecyl acrylate, which undergo expansion upon oil absorption, facilitating deformation and expansion. Nonetheless, despite these advances, the application of the aforementioned resin-based plugging agents in OBDF systems on-site is limited by high production costs and a lack of universality, hindering optimal compatibility. However, the above research results used as plugging agents, didn't discuss the contradiction between the dispersibility of plugging agents and their staying capacity in the loss channel. In this study, the plugging agent prepared by us can be dispersed in oil-based drilling fluids due to its lipophilicity. Additionally, it can also be seen that the plugging agent has certain staying capacity in the plugging experiment. Therefore, the resin prepared in this study is of great significance for the plugging problems encountered during oilfield development.

In this paper, the study has developed a novel high-temperature-resistant suspension polymerization resin (MMA-SMA-St) tailored for OBDF applications. The resin is synthesized using methyl methacrylate (MMA), stearyl methacrylate (SMA), and styrene (St) as monomers. Incorporating methyl methacrylate and styrene addresses the issue of flexible crosslinking instability, which is attributed to the lengthy carbon chain present in stearyl methacrylate. Furthermore, the employment of suspension polymerization offers a practical framework for the field application of this plugging material.

## Experimental

### Materials

Methyl methacrylate (MMA, 89 wt%), stearyl methacrylate (SMA, 90 wt%), polyvinyl alcohol (PVA, 87–89 wt%), and styrene (St, 99.5 wt%) were sourced from Aladdin Industrial Co., Ltd, China. *N*,*N*-Methylenebisacrylamide (MBA, 99 wt%) was obtained from Sinopharm Chemical Reagent Co., Ltd., China. Benzoyl peroxide (BPO, 99 wt%) and ethyl acetate (EAC, 99 wt%) were procured from Macklin Biochemical Co., Ltd, Shanghai, China. Styrene was purified using a 20% sodium hydroxide solution and subsequently neutralized with deionized water. No further purification was applied to the other chemicals. Deionized water was produced in-house.

### Synthesis of oil absorbing expansion resin

A volume of 120 mL deionized water was transferred into a 250 mL four-neck flask and mixed with PVA. This mixture was stirred at room temperature until the PVA was fully swollen, then heated to 80 °C and stirred for 30 minutes to ensure complete dissolution of PVA, followed by cooling to 50 °C. Monomers MMA and SMA, cross-linker MBA (0.6 g), and pore-forming agent EAC (4.5 g) were combined and stirred for 15 minutes, yielding solution A. Separately, the rigid monomer St and initiator BPO were stirred together for 15 minutes, forming solution B. Both solutions A and B were then stirred together for 15 minutes. Solution A was added to the pre-prepared PVA solution under a nitrogen atmosphere. After stirring the mixture for 30 minutes, the temperature was increased to 85 °C, and solution B was added. The reaction mixture was stirred continuously for 8 hours, then cooled to room temperature, and filtered to collect transparent resin microspheres of irregular sizes. The microspheres were washed thrice with hot deionized water and ethanol, then dried in a vacuum oven at 80 °C for 24 hours.

### Oil absorption and expansion performance

The oil absorption and expansion rate of the resin are crucial for the formulation of oil-based drilling fluid systems and the prediction of their plugging efficacy. The oil absorbency (W) of the product was quantified using a gravimetric method. A pre-weighed 1 g sample of the product was enclosed in a filter bag and immersed in oil at room temperature. For complete oil absorption, a duration of 24 hours was required. Subsequently, the filter bag containing the product was extracted from the oil and permitted to drain for one minute. Immediately after draining, the product was removed from the filter bag and weighed. Oil absorbency was calculated using the equation below:1
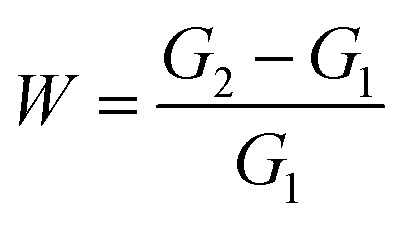


The oil absorbency is calculated using the formula wherein *G*_1_ and *G*_2_ represent the weights of the resin before and after oil absorption, respectively. Unless otherwise specified, “oil absorbency” refers to the total oil absorbency.^24^

### Preparation of OBDF

As noted by Shen *et al.*^25^ taking into account both the stability and the cost of the OBDF, 3# white oil was chosen as the base oil with an oil-water ratio of 80 : 20. The detailed composition of the drilling fluid is presented in Table 1. In this context, the amide group within the non-ionic emulsifier, namely fatty acid polyamide, serves as a hydrophobic entity oriented towards the oil phase, whereas the amino group functions as a hydrophilic entity directed towards the water phase. This configuration effectively reduces the free energy at the oil–water interface, thereby enhancing the stability of the drilling fluid mud. Organic clay, characterized as an oleophilic material, is derived from hydrophilic bentonite modified with dodecyltrimethylammonium bromide (DTAB). To negate the effects of filtrate reducers on the filtration rate, the system is formulated without the addition of any filtrate reducer.

**Table 1:** The specific drilling fluid formula

Reagent	Dosage	Reagent action
3# white oil	240 g	Base oil
Fatty acid polyamide	9 g	Emulsifier
Organic soil	9 g	Leaf cutting agent, tackifier
CaO	9 g	Alkalinity regulator
25% CaCl_2_	60 mL	Adjusting the oil–water ratio
Barite (4.2 g cm^−3^)	211 g	Weighting agent

### Evaluation of drilling fluid performance

#### Plugging experiment

To accurately assess the sealing efficacy of plugging materials on fractures, the study utilized a custom-built simulation fracture apparatus in conjunction with a high-temperature and high-pressure (HTHP) filtration instrument (GGS42, Qingdao Hengtaida Electromechanical Equipment Co., Ltd.) for evaluation. The process began with the assembly of a micro fracture simulation rigid seam board, consisting of two semi-cylindrical sections with an external diameter of 54.3 mm. The fracture width was set by inserting aluminum foil strips of varying thickness (20–1000 μm), adjusted to specific widths (*x* = 0.25 μm, 0.5 μm, 0.75 μm), and secured with nitrile rubber seals. The assembled simulation fracture plate was then placed at the lower end of the HTHP filtration instrument's cylinder, followed by the addition of the pre-mixed drilling fluid slurry into the device. The experiment was conducted by setting the HTHP filtration instrument to a temperature of 160 °C, simulating high-temperature and high-pressure conditions.

#### Sand bed experiment

Prepare an OBDF slurry containing *x*% (*x* = 2, 4, 6) of the resin plugging agent and subject it to aging in an oven at 160 °C for 16 hours. Subsequently, weigh 350 cm^3^ of quartz sand within a specified mesh size, thoroughly wash and dry it, then transfer it into the cylindrical chamber of a medium-pressure sand bed filter. Level and compact the sand uniformly before gently introducing 250 mL of the aged OBDF slurry enriched with a designated concentration of the plugging agent. After sealing the setup, apply pressure up to 0.7 MPa. Monitor the penetration and filtration of the bentonite slurry within the sand layer, and ascertain the depth of filtrate penetration into the sand bed after a duration of 30 minutes.

#### Electrical stability test

According to,^26^ the electrical stability (ES) of OBDF is an indicator of their emulsion stability and oil wettability. Therefore, measuring the ES values before and after the addition of resin provides a direct assessment of the plugging agent's effect on the drilling fluid's stability. The procedure involves stirring the prepared drilling fluid for 15 minutes, then inserting the motor probe into the fluid to obtain a reading. Given the intricate influence of the drilling fluid's chemical composition on its ES, it is essential to conduct multiple measurements of the drilling fluid's ES and calculate the average value.

#### Rheological test

The rheological properties of resin were measured by Haake Mars 60 rheometer at 50 °C.

The rheological parameters of OBDF, such as the apparent viscosity (AV), plastic viscosity (PV), and yield point (YP) of OBDF, were determined through measurements of the viscosities at two shear rates of 600 rpm and 300 rpm at room temperature by using a ZNN-D6L rotational viscometer (Qingdao, China). Calculated from the values of *Ø*_600_ and *Ø*_300_ by the following formulas:^14^

2 Apparemt viscosity (AV) = 0.5*Ø*_600_ (mPa s)

3 Plastic viscosity (PV) = *Ø*_600_ − *Ø*_300_ (mPa s)

4 Yield point (YP) = 0.511(2*Ø*_300_ − *Ø*_600_) (Pa)

where the *Ø*_600_ and *Ø*_300_ represent the reading on the rheometer dial at speeds of 600 and 300, respectively.

### Characterizations

#### Contact angle analysis

The contact angle of the resin was observed by an optical contact angle meter (KRUSS DSA30S, Germany). A smooth thin film was obtained by pressing purified and dried samples for 5 min under 10 MPa at room temperature. Then, the contact angle between white oil and film under air conditions was measured.

#### X-ray photoelectron spectrometry analysis

The elements of the sample were verified using X-ray photoelectron spectroscopy (XPS, Thermo Scientific ESCALAB 250Xi VG).

#### Fourier transform infrared spectroscopy (FT-IR) test

The oil-absorbing resin underwent analysis through Fourier transform infrared spectroscopy (FT-IR; model: Nicolet iS 10), encompassing 4 to 32 scans per millisecond across a scanning wavenumber spectrum of 4000 to 400 cm^−1^.

#### TGA-DSC measurements

The thermal properties of the resin were determined using a thermal analysis apparatus (TGA/DSC 3+/1600 HT, METTLER TOLEDO) under nitrogen flow, with the temperature range set from 30 to 800 °C at a heating rate of 10 °C min^−1^.

#### Scanning electron microscopy analysis

Scanning electron microscopy (SEM) analyses were measured by a scanning electron microscope (Phenom ProX, thermos scientific). First, a specific quantity of resin was adhered to the glass slide to quantify the look of the material. The slide was then attached to the conductive adhesive after being cured under vacuum for 30 minutes. Lastly, the resin was lightly coated with gold before being seen under a SEM. Scanning electron microscopy (SEM) analysis was conducted using a scanning electron microscope (Phenom ProX, Thermo Scientific). Initially, a defined quantity of resin was adhered to a glass slide to facilitate the examination of the material's appearance. Subsequently, the slide was affixed to conductive adhesive following a 30 minutes vacuum curing process. Finally, the resin underwent a light gold sputtering to enhance its conductivity before SEM observation.

#### Crosslinking degree analysis

The crosslinking degree of the resin were verified using a field nuclear magnetic resonance analyzer (VTMR20-010V-I, SuZhou). The resonance frequency is 22.00 MHz, the temperature of the magnet is controlled at 35.00 ± 0.01 °C, and the diameter of the probe coil is 10 mm. The XLD-3 inversion model was used to fit the crosslinking density parameters. The *T*_2A_ (relaxation time of the signals from the cross-linked portion), *T*_2B_ (relaxation time of the signals from the uncrosslinked portion), and crosslinking density were obtained by testing.

## Results and discussion

### Synthetic mechanism of the oil-absorbing resin

**Fig. 1:**
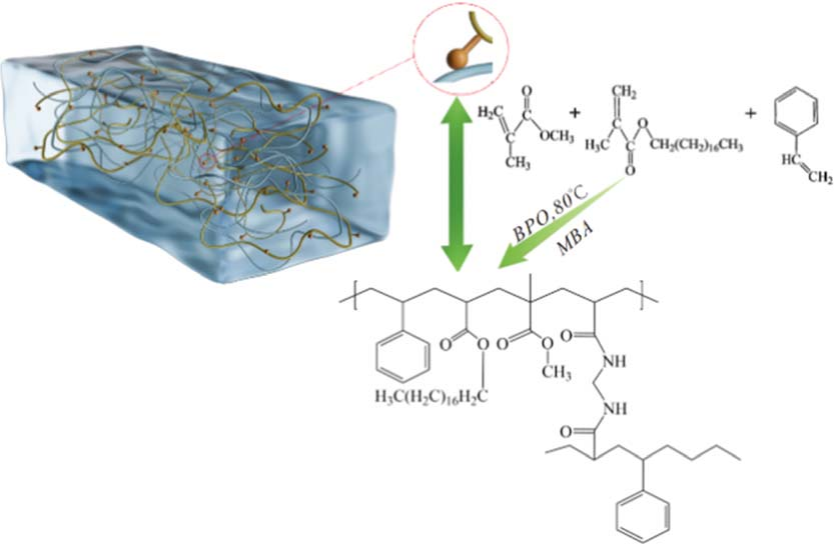
Diagram of the synthetic mechanism of the oil-absorbing resin.

Fig. 1 presents the schematic representation of the synthetic mechanism for the oil-absorbing resin. In the ternary copolymer synthesis, methyl methacrylate functions as a hard monomer, octadecyl methacrylate serves as a flexible macromolecule due to its long-chain ester composition, and styrene acts as a rigid molecule enhancing the resin's resistance to pressure. All three monomers contain C=C bonds, allowing them, under the influence of an initiator and a cross-linking agent, to not only undergo bulk polymerization but also to create a three-dimensional network with a structured framework.^27,28^ Benzoyl peroxide facilitates polymerization by generating benzoyl radicals through thermal decomposition, and *N*,*N*′-methylenebisacrylamide (MBA) engages in cross-linking and polymerization *via* its dual C=C bonds. Furthermore, during the polymerization process, the aforementioned monomer free radicals contribute to the generation of additional chain free radicals, fostering the development of cross-linked networks.^29,30^ While the benzene ring in styrene reduces the polymer's crosslinking density, the long carbon chain in octadecyl methacrylate increases it. This combination achieves a balance between oil absorption capacity and polymer rigidity. Additionally, incorporating ethyl acetate creates a porous cross-linked structure on the resin's surface, which enhances its oil absorption capabilities.^31^

### Formula screening of oil-absorbing resin

**Fig. 2:**
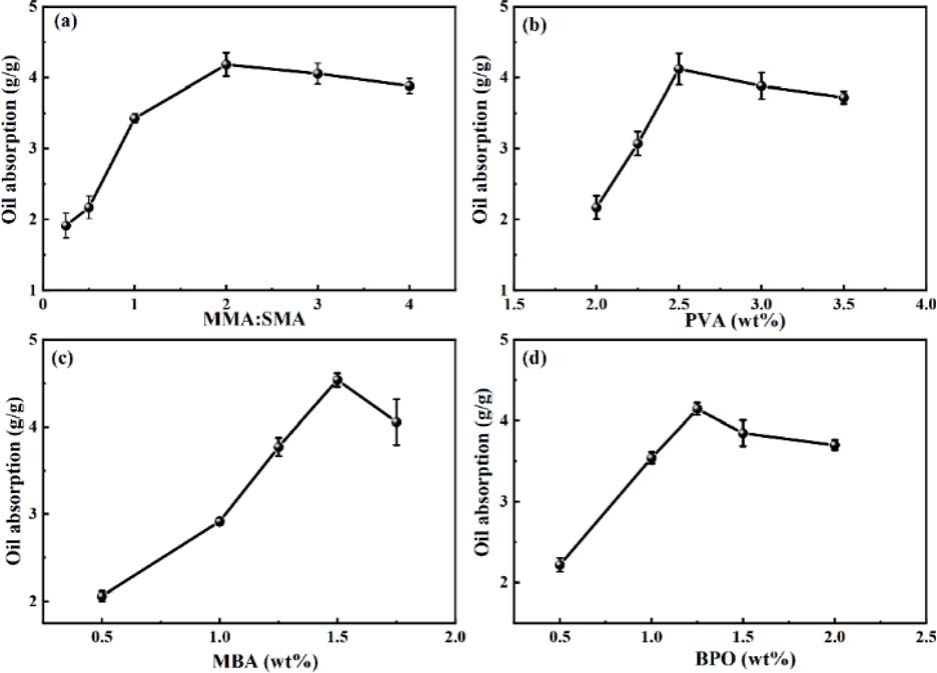
The oil absorption expansion rate of the resin in different conditions: (a) the effects of different monomer ratios on oil absorption of the resin; (b) the effect of PVA on absorption of the resin; (c) the effect of MBA on absorption of the resin; (d) the effect of BPO on absorption of the resin.

Fig. 2 illustrates the oil absorption expansion rate of the resin under various conditions. The effects of dispersant PVA, cross-linker MBA, initiator BPO, and the ratio of MMA to SMA on the resin's oil absorption properties were investigated. Throughout this experiment, the total mass of monomers remained constant, with the amounts of PVA, MBA, and BPO specified as a percentage of their mass relative to the total monomer mass.

The presence of multiple side chain groups significantly influences the oil absorption capacity of resins. This phenomenon is attributed to compounds with multi-side chain structures expanding the internal volume of the resin *via* crosslinking agents. Consequently, when oil molecules penetrate the resin, the interaction surface between the resin and oil molecules enlarges, leading to an increased resin expansion rate. Thus, the proportions of MMA and SMA monomers utilized in the synthesis critically affect the resin's oil absorption expansion rate. Fig. 2(a) demonstrates that as the MMA : SMA molar ratio escalates, the oil-absorbing capability of the resin progressively improves. It is observed that an augmentation in MMA correlates with a heightened oil absorption rate. Furthermore, the oil absorption rate peaks when the MMA : SMA molar ratio reaches 2 : 1.^18^ Below a 2 : 1 MMA : SMA molar ratio, the presence of SMA yields numerous cross-linking points within the resin. However, an excess of these points restricts the flexibility of chain segments in the resin, diminishing the oil absorption expansion rate. Conversely, when the ratio exceeds 2 : 1, a reduced SMA concentration leads to a scarcity of long alkyl side chains in the polymer, thereby decreasing oil absorption capability. Fig. 2(b) illustrates the impact of varying monomer ratios on the resin's oil absorption capacity, revealing an initial increase followed by a decrease in oil absorption with the rise in PVA concentration, peaking at a 2.5% PVA concentration. At low PVA concentrations, oil-soluble monomers experience inadequate dispersion in water, leading to monomer aggregation, increased impurities within the polymer, and a reduced oil absorption expansion rate. Conversely, excessively high concentrations of PVA result in over-dispersion of monomers, which adversely affects the conditions conducive to polymerization, thereby diminishing the oil absorption rate.

Fig. 2(c) depicts the curve correlating the content of cross-linker *N*,*N*′-methylenebisacrylamide with the resin's oil absorption capacity. At lower MBA concentrations, the polymer exhibits fewer cross-linking points, resulting in a less effective cross-linking network within the resin. This insufficiency was observed during experiments where a minimal cross-linking agent concentration resulted in a product that was turbid and challenging to purify. As illustrated in Fig. 2(c), a progressive increase in MBA concentration led to the formation of a densely cross-linked structure within the resin. This structure facilitates oil molecule ingress *via* capillary action, with increasing numbers of oil molecules causing cross-linking points to rupture, allowing the long alkyl side chains within the resin to extend and the resin to absorb oil and swell. Furthermore, a higher degree of cross-linking enhances the resin's oil storage capacity, thereby augmenting its expansion rate, as indicated by Zhang's research.^32^ The optimal oil absorption rate was observed at the MBA concentration of 1.5%; beyond this point, the oil absorption rate declined. This decrease is attributed to higher cross-linking agent concentrations resulting in shorter chain segments between cross-linking points, reducing their mobility and, consequently, the resin's swelling capability.

Fig. 2(d) illustrates the influence of benzoyl peroxide (BPO) on the oil absorption characteristics of the resin, with the oil absorption expansion ratio peaking at a BPO concentration of 1.25%. Given the employment of oil-soluble monomers in the synthesis process, the oil-soluble free radical initiator BPO is utilized accordingly. BPO is recognized as an effective initiator for the synthesis of acrylic ester polymers.^33^ When the amount of BPO was low, the internal crosslinking degree of the resin was small, the contact area between oil molecules and polymers was also small, and the oil absorption rate decreased. The effect of initiators on the crosslinking degree of polymers had a peak. When there were too many initiators, not only would they not increase the oil absorption rate of the resin, but they would also affect the formation of polymer crosslinking structure and reduce the oil absorption rate of the resin.

### Structure evaluation of oil-absorbing resin

**Fig. 3:**
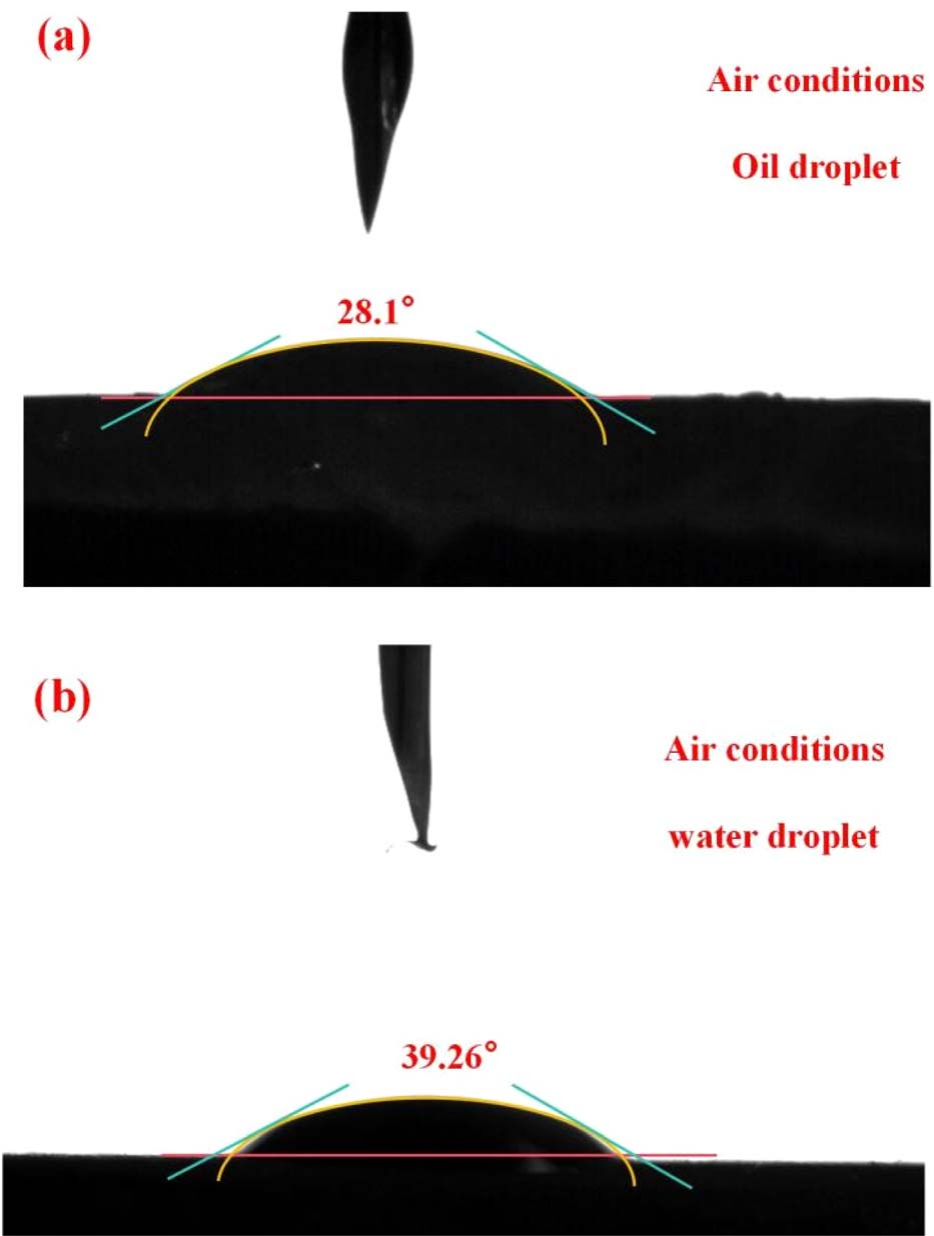
The results of contact angle: (a) the contact angle of white oil on the resin film surface under air conditions; (b) the contact angle of water on the resin film surface under air conditions.

The contact angle of the oil droplet on the resin film surface under air condition was 28.1° shown in Fig. 3(a), and the contact angle of the water droplet on the resin film surface under air condition was 39.26° shown in Fig. 3(b), indicating that the resin film was both oleophilic and hydrophilic. On the one hand, there are benzene rings (from styrene) on the surface of the resin, and on the other hand, the long carbon chain in SMA also makes the resin exhibit lipophilicity. In addition, the hydrophilicity of the resin is due to the addition of hydrophilic crosslinking agent MBA, where the acrylamide group makes the resin hydrophilic.

**Fig. 4:**
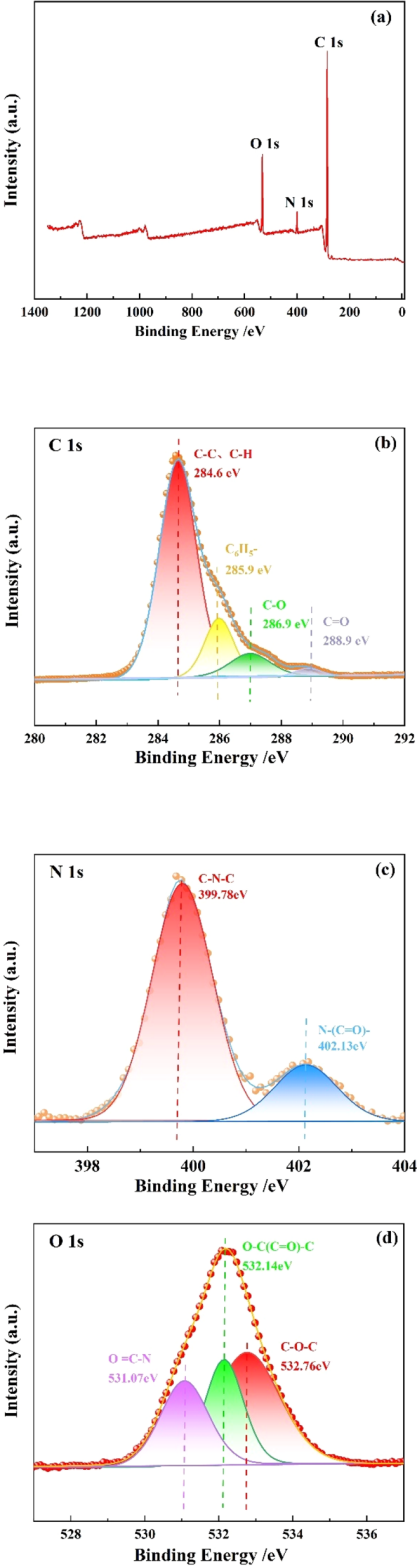
The results of XPS: (a) survey scan of the resin; (b) the high-resolution XPS spectrum of C 1s of the resin; (c) the high-resolution XPS spectrum of N 1s of the resin; (d) the high-resolution XPS spectrum of O 1s of the resin.

The resin structure was investigated using XPS, and the results are shown in Fig. 4. As shown in Fig. 4(a), the resin contains three elements: C, N, and O, and the characteristic peak intensity of C element is the highest, while N element is the lowest. Fig. 4(b) shows the high-resolution XPS spectrum of C 1s of the resin. After deconvolution, four peaks can be found in the resin, the peaks with binding energy of 288.9 and 286.9 eV are C=O and C–O, indicating that the resin contains SMA and MMA. The peak with a binding energy of 285.9 eV is C_6_H_5_^−^, indicating the presence of St. In addition, the peak representing C=C was not found in the C 1s spectrum, indicating that the product does not contain reactive monomers. Fig. 4(c) shows the high-resolution XPS spectrum of N 1s of the resin. There were two peaks found in the resin. The peaks with binding energy of 402.13 and 399.78 eV are N–(C=O)– and C–N–C, representing the presence of MBA. Fig. 4(d) shows the high-resolution XPS spectrum of O 1s of the resin. After deconvolution, three peaks can be observed in the resin, representing C–O–C, O–(C=O)–C and O=C–N. C–O–C, O–(C=O)–C indicate the presence of SMA and MMA in the resin. The presence of O=C–N indicates the containment of crosslinking agent MBA in the resin.

**Fig. 5:**
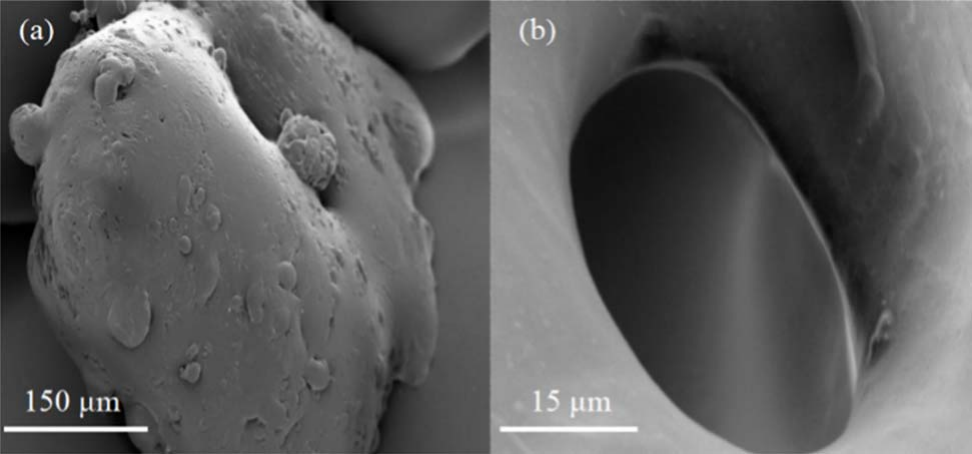
SEM picture of the resin: (a) SEM pictures of the resin magnified 1000 times; (b) SEM pictures of the resin magnified 10 000 times.

Fig. 5(a) displays scanning electron microscope (SEM) images of the resin at 1000× magnification, revealing an irregular surface adorned with numerous gully-like structures. This uneven topology augments the contact surface between the resin and oil molecules, significantly enhancing the oil absorption expansion rate. Fig. 5(b) provides an SEM image of the resin at 100 00× magnification, showcasing the presence of round microporous structures on the surface. These micropores facilitate the penetration of oil molecules into the resin's interior, further extending the contact surface between them, thereby augmenting the oil absorption expansion rate.

**Fig. 6:**
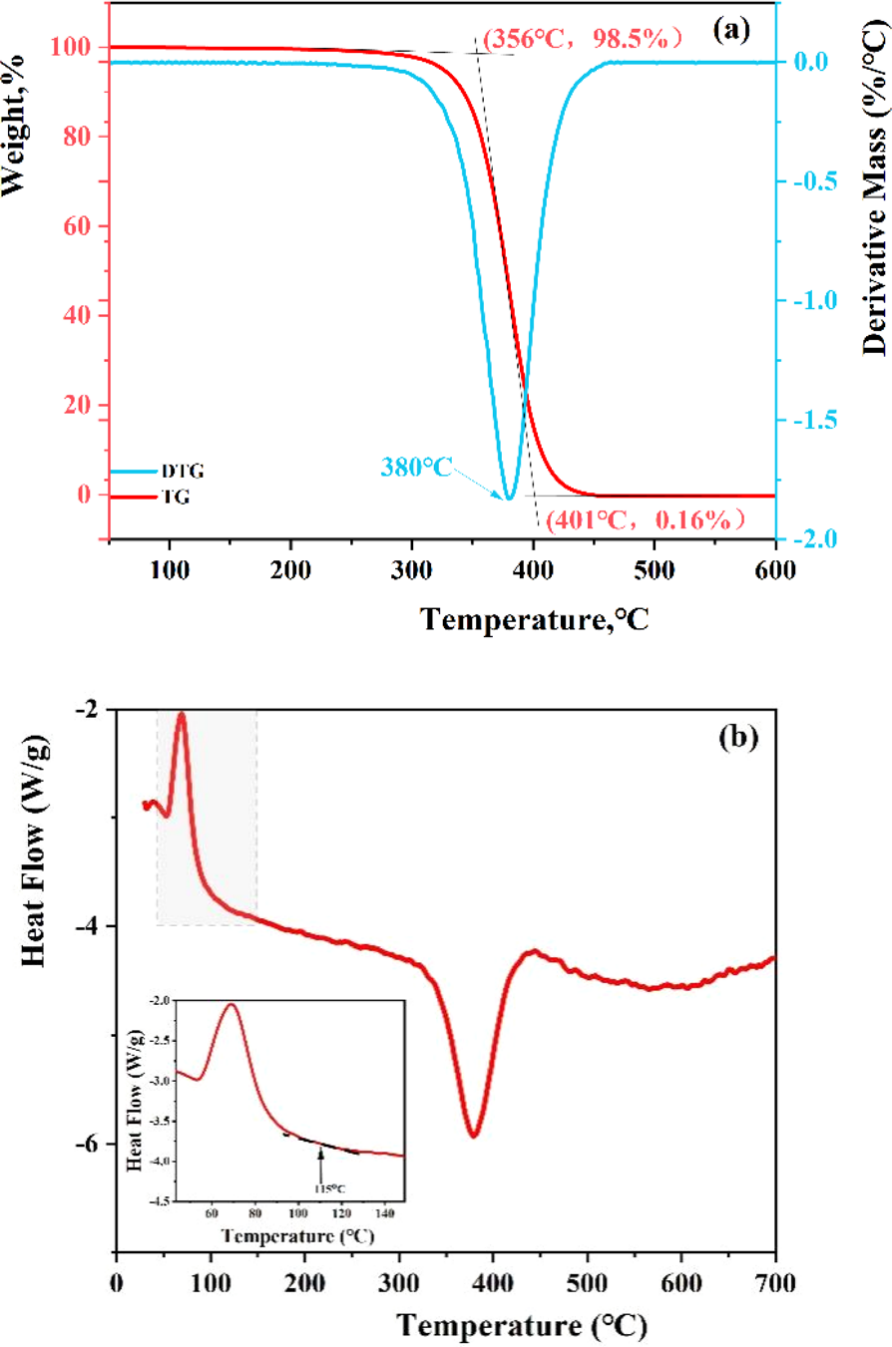
TGA-DSC analysis of the resin: (a) TGA-DTG curve of the resin; (b) DSC curve of the resin.

The polymer's molecular structure was examined using thermogravimetric analysis (TGA) coupled with differential scanning calorimetry (DSC). As depicted in Fig. 6(a), the resin's weight reduction occurs in three distinct phases. The initial phase of weight loss extends from room temperature to 356 °C, during which the TGA curve maintains a nearly constant slope. The weight loss rate up to 356 °C is minimal, at only 1.5%, attributed to the volatilization of solvents in the resin. In the subsequent phase, spanning 356–401 °C, the resin's weight decreases significantly, with the DTG (differential thermogravimetric) curve peaking at 380 °C, marking the onset of thermal degradation of the resins. At 401 °C, the residue is a mere 0.16%, indicating a high resin purity. Beyond 414 °C in the final phase, the resin transitions to carbon, and the TGA curve stabilizes, demonstrating the resin's commendable thermal stability. These results align closely with the TGA findings for nano-sized poly(styrene-lauryl acrylate),^34^ showcasing even greater purity. In addition, the glass transition temperature (*T*_g_) was characterized by DSC shown in Fig. 6(b). According to the curve, the softening point of resin is 115 °C. The glass transition temperature (*T*_g_) refers to the temperature corresponding to the transition from a glassy state to a highly elastic state. Most of the cracks that cause leakage are located in deep formations, where the temperature is generally above 100 °C. Therefore, when the resin enters the formation exceeds the *T*_g_, the resin will transform into a high elastic state and enter the cracks due to the positive pressure difference. After oil absorption, the resin will expand to seal the leakage cracks.

**Fig. 7:**
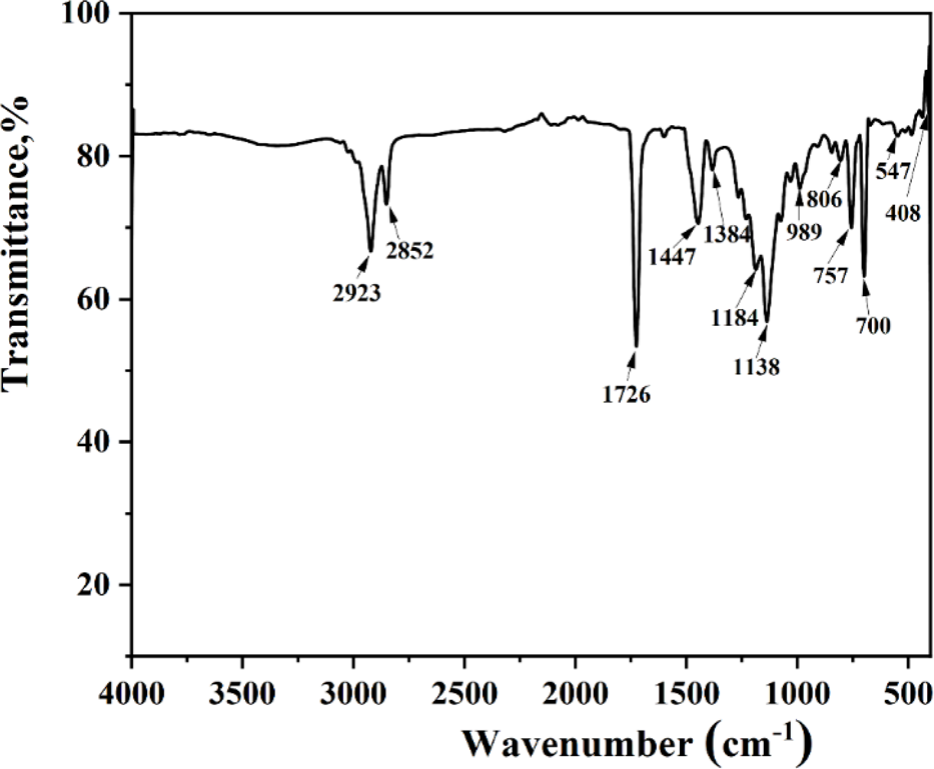
FTIR spectrum of the resin.

The infrared (IR) spectroscopy results of the oil-absorbing resin are depicted in Fig. 7. Absorption peaks at 2923 and 2852 cm^−1^ correspond to the asymmetric and symmetric stretching vibrations of C–H in the methyl and methylene groups, respectively, alongside –CH.^18^ The peak at 1726 cm^−1^ is associated with the stretching vibration of the acrylate ester carbonyl (C=O), denoting the incorporation of MMA chain segments within the resin. Peaks at 1447 and 1383 cm^−1^ are ascribed to the C=C stretching vibrations in the benzene ring. Additionally, peaks at 806, 757, and 700 cm^−1^ are linked to the out-of-plane bending vibrations of hydrogen in the benzene ring, characteristic of monosubstituted benzene.^34^ The presence of styrene-acrylonitrile (SMA) chain segments is evidenced by peaks at 1187 and 1138 cm^−1^, representing the C–O bond stretching vibrations, and a peak at 989 cm^−1^, associated with the in-plane rocking vibration of –CH in the long-chain methylene group. Collectively, the IR spectroscopy results confirm the involvement of MMA, SMA, and styrene (St) in constructing the resin's skeletal structure. Furthermore, the absence of C=C absorption peaks in the range of 1640–1675 cm^−1^ suggests a comprehensive synthesis reaction, where the unsaturated groups of the three monomers have engaged in grafting, leading to the formation of a three-dimensional network structure.

The results of the model rubber crosslinking density test are shown in Table 2. According to the result, the relaxation time of crosslinked signals is very short, while the relaxation time of the signals from the uncrosslinked portion is very long. The relaxation time represents the degree of freedom of hydrogen protons in the resin, indicating a high degree of resin cross-linking. In addition, the cross-linking density was 76.03 × 10^−4^ mol mL^−1^, indicating the formation of an internal cross-linking network structure in the resin. To a certain extent, the higher the cross-linking density of the resin, the more cross-linking bonds there are per unit volume of the resin, and the greater the degree of cross-linking. When oil molecules enter the interior of the resin, the cross-linking network is stretched by the oil molecules, causing the resin volume to increase and the oil absorption rate to increase.

**Table 2:** The result of the model rubber crosslinking density test

*T*_2A_ (ms)	*T*_2B_ (ms)	Crosslinking density (10^−4^ mol mL^−1^)
0.13	1.27	76.13

Table 3 presents the outcomes of the electrical stability and rheological tests. These tests evaluated the rheological properties and demulsification voltage of drilling fluids before and after the incorporation of 3% resin, under various temperatures and aging conditions. Relative to drilling fluids devoid of resin, the apparent viscosity (AV), plastic viscosity (PV), and yield point (YP) exhibited increases both before and after aging. This rise is attributed to the addition of solid resin particles, which heightens the flow friction resistance among solids and liquids, as well as between the solids and liquids themselves. Furthermore, the introduction of resin particles markedly elevates the demulsification voltage, a consequence of the enhanced viscosity of the drilling fluid imparted by the resin particles, leading to a more stable emulsion and, hence, an increased demulsification voltage. Additionally, as illustrated in Table 3, the aging temperature's elevation significantly affects the rheological properties and demulsification voltage of the drilling fluid mud, suggesting commendable compatibility between the resin and mud.

**Table 3:** The results of electrical stability test and rheological test

Type	Aging temperature (°C)	Condition	AV (mPa s)	PV (mPa s)	YP (Pa)	*E*_s_ (V)
Original OBDF	120	Before aging	64	62	2	550
		After aging	66	63	3	541
Original OBDF	140	Before aging	62	58	4	544
		After aging	65	61	4	542
Original OBDF	160	Before aging	60	57	3	540
		After aging	58	54	4	533
With 3% resin	120	Before aging	89	80	9	1290
		After aging	87	81	6	1287
With 3% resin	140	Before aging	87	81	6	1256
0		After aging	88	83	5	1198
With 3% resin	160	Before aging	84	80	4	1100
		After aging	86	82	4	1088

### Oil absorption performance of the resin

#### Effect of absorption time

**Fig. 8:**
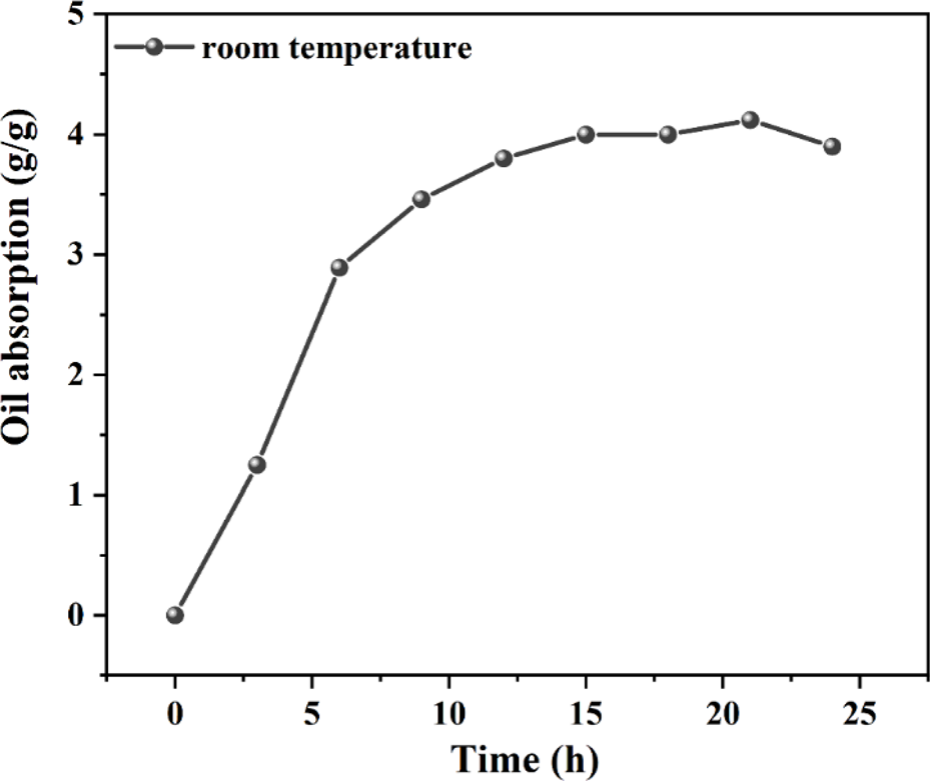
Oil absorption performance of the high-oil-absorbing resin under different time conditions.

Fig. 8 illustrates the resin swelling process over 24 h, segmented into four distinct stages. Initially (0–6 h), the resin's swelling rate is notably swift, with a discernible slowdown in the swelling rate within the first hour of resin and oil molecule interaction. Subsequently, after this hour, there is a significant acceleration in the swelling rate. This acceleration then diminishes in the second phase (6–15 h), culminating in a near cessation of swelling in the final phase (15–24 h) as the resin approaches swelling equilibrium. Notably, the last 3 hours witness a reduction in swelling rate. In the initial stage of oil absorption, a fraction of oil molecules penetrate the interior of the resin molecules through capillary action, leading to a relatively slow oil absorption rate within the first hour. Following this period, the oil molecules achieve comprehensive contact with the resin particles, transitioning the control of oil absorption from molecular diffusion to thermodynamics. At this period, sufficient oil molecules have entered the resin particles' interior, facilitated by van der Waals forces.^18^ The resin's flexible macromolecular chain segments begin to expand, engaging in solvation with the oil molecules. This stage is marked by the highest oil absorption rate, driven by the resin's internal three-dimensional network composed of covalent bonding crosslinking points, physical crosslinking regions, and entanglement connections.^35^ As the flexible macromolecular chains unfold, the majority of oil molecules infiltrate the resin, slowing the diffusion rate of oil molecules within the resin. Consequently, during this third stage, the resin's oil absorption rate decelerates under dynamic control. Ultimately, as the thermodynamic driving force and network elastic retraction force equilibrate, the resin achieves saturation swelling. Therefore, in the final stage, the oil absorption rate of the resin essentially stabilizes.

#### Effect of temperature

The resin was incorporated into nonwovens and encapsulated within a tube resistant to temperature and pressure, then subjected to conditions at room temperature and at 80, 120, 140, and 160 °C, respectively, immersed in 3# white oil. To determine the oil absorption swelling capability, the mass ratios before and after oil absorption were evaluated using gel particles with a particle size of 0.1 mm.

**Fig. 9:**
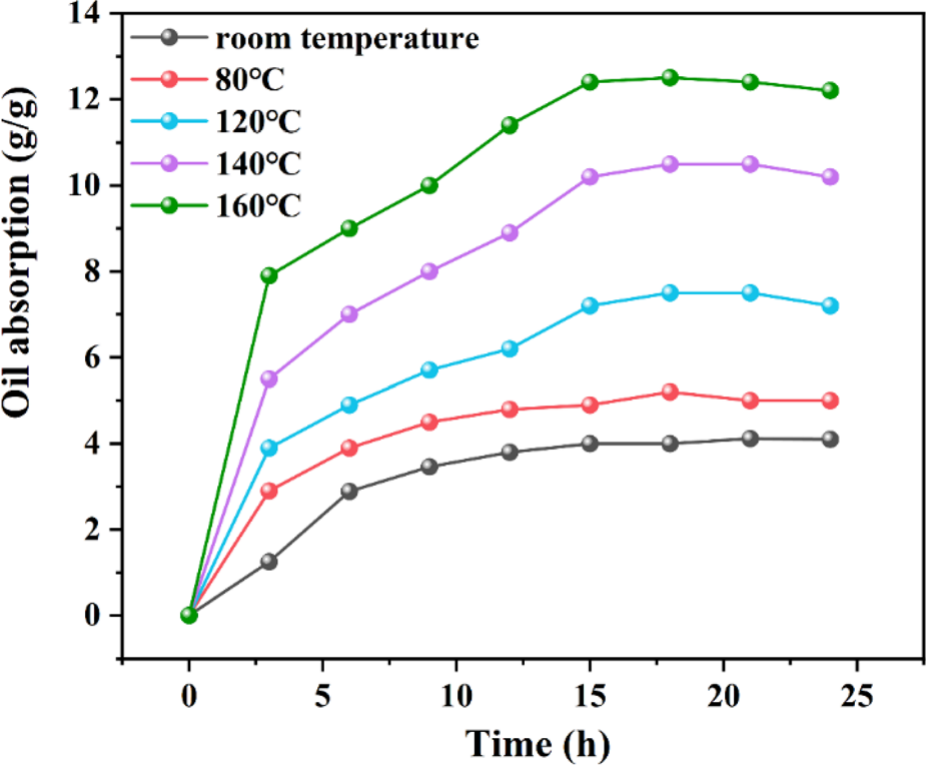
Curves for the oil absorption ratios of the resin at different temperatures.

Fig. 9 displays the experimental data on the oil absorption expansion rate of the resin at room temperature, as well as at 80, 120, 140, and 160 °C. Notably, at 160 °C, the resin's oil absorption expansion ratio reached 12.2. Research of the resin's oil absorption process at room temperature revealed that the resin's maximum oil absorption rate is governed by thermodynamics. Additionally, the resin's oil absorption saturation rate is primarily influenced by its crosslinking density and the solvation interactions between oil molecules and the resin's oleophilic groups. Consequently, with rising temperatures, the initial stage of oil absorption, dominated by molecular diffusion, facilitates greater contact between oil molecules and the resin surface, thereby increasing the number of oil molecules penetrating the resin's interior. In the subsequent stage, dominated by thermodynamic control, the interaction between oil molecules and the resin's oleophilic groups becomes more comprehensive, enhancing the solvation effect and thus the resin's expansion volume.

### Plugging performance of resin

#### Plugging experiment

The outcomes of the crack plugging experiment are detailed in Table 4. Without the addition of resin particles, OBDF exhibits inadequate sealing efficacy across three different crack sizes, resulting in total fluid loss when the crack width reaches 0.75 μm. Conversely, the incorporation of 3% resin particles effectively seals small cracks and substantially mitigates leakage through slightly larger fissures. The plugging efficiency remains relatively consistent before and after aging, underscoring the prepared resin particle plugging agent's robust stability. Furthermore, observations during the experiment revealed that the resin particles remained well-dispersed within the mud, without any signs of agglomeration or clumping, demonstrating excellent compatibility between the resin particles and the drilling mud.

**Table 4:** The crack plugging experiment

Type	Slit width (μm)	Condition	FL_HTHP_ (mL)
Original OBDF	0.25	Before aging	5
		After aging	7
Original OBDF	0.5	Before aging	10
		After aging	10
Original OBDF	0.75	Before aging	Full loss
		After aging	Full loss
With 3% resin	0.25	Before aging	0
		After aging	0
With 3% resin	0.5	Before aging	0
		After aging	1
With 3% resin	0.75	Before aging	2
		After aging	2.6

**Fig. 10:**
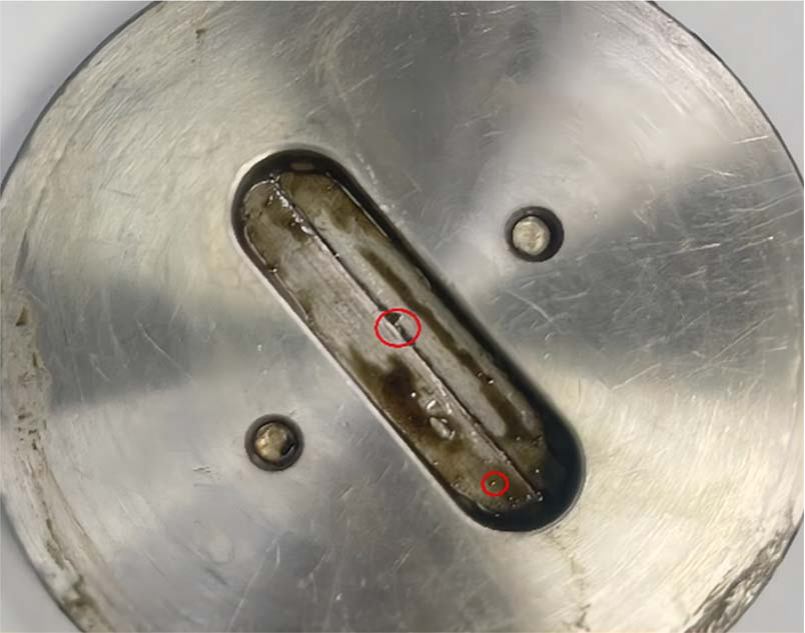
The condition of the crack board in the plugging experiment of the crack width is 0.25 μm.

Fig. 10 illustrates the state of the crack plate during the plugging experiment with a crack width of 0.25 μm. The regions highlighted in red represent resin particles. Observation reveals that resin particles accumulate within the crack. Subject to high temperature and pressure conditions, these particles undergo deformation and penetrate the crack under compressive forces. Owing to the resin's expansion and viscosity within the environment of oil molecules, it remains lodged within the crack, thereby effectively sealing the leak.

### Sand bed experiment

Table 5 presents the results of sand bed experiments conducted with OBDF mud containing varying concentrations of resin-based plugging agents. The data indicate that as the concentration of resin particles is increased, the depth of intrusion into the sand bed significantly diminishes.

**Table 5:** Sand bed experimental results of OBDF mud with different concentrations of resin

Component	Testing conditions	Invasion depth (cm)	Lost circulation (cm)
2% resin	30 min, 0.7 MPa	3.8	Zero
4% resin	30 min, 0.7 MPa	1.3	Zero
6% resin	30 min, 0.7 MPa	0.6	Zero

Traditional plugging materials often necessitate specific particle sizes to effectively seal lost circulation zones. In scenarios involving smaller void spaces, these rigid plugging materials must be crushed and resized before incorporation into the plugging mud. Conversely, the resin-based plugging materials developed in this study are capable of adapting to various pore sizes in loss channels, thanks to their inherent variability. Furthermore, the incorporation of styrene (St), with its benzene ring structure, imparts a degree of pressure resistance to the mixture. Beyond this, the resin particles exhibit a certain viscosity upon oil absorption, allowing them to persist within the leakage channels. This capacity enables them to form a pressure-sealing layer possessing adequate strength to mitigate fluid loss effectively.

### Sealing mechanism

**Fig. 11:**
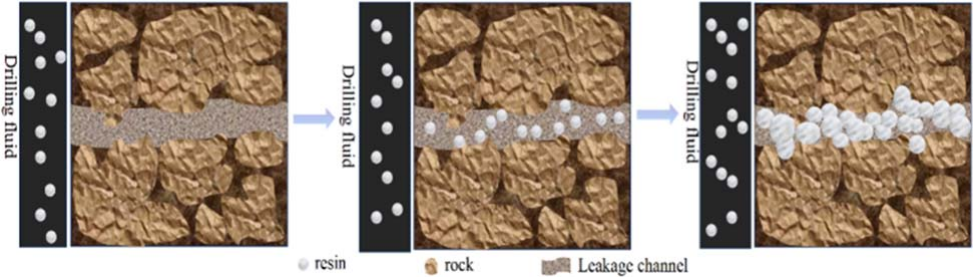
The schematic diagram of resin entering the leakage channel to block the leak after a well leakage occurs underground.

Fig. 11 depicts a schematic representation of resin particles entering a leakage channel to seal a leak after an underground well breach occurs. The resin particles, owing to their excellent deformability, navigate into the leakage channel. Accumulation of a substantial number of resin particles within the channel counterbalances the drilling fluid column's pressure with the channel's internal pressure, thereby achieving effective fill.^36^ Over time, as the resin particles remain in the drilling fluid, they absorb oil and swell, thereby fulfilling the leak plugging objective. It is important to note that most plugging materials under study require favorable dispersibility in the oil phase, necessitating the synthesis of materials rich in lipophilic groups. However, the challenge of material retention within the leaking stratum is often overlooked. This oversight stems from the fact that upon the entry of OBDF into the formation, the rock surface in contact with the drilling fluid transitions to an oil-wet surface, which impedes the plugging material's adherence to the leaking wall.^37^ In response, our synthesis of the resin incorporates the water-based crosslinking agent MBA. Additionally, we minimize the use of styrene-acrylonitrile (SMA) with its lengthy carbon chains. To enhance the resin's pressure resistance and augment the addition of styrene (St), our findings indicate that the resin material synthesized under these parameters exhibits superior plugging performance in such scenarios.^38^

## Conclusions

(1) A ternary self-swelling oil-absorbing resin was synthesized using polymeric monomers of St, SMA, and MMA. BPO served as the initiator, MBA as the cross-linker, and EAC as the pore-forming agent.

(2) The molecular structure and characteristics of the resin were investigated using SEM, TG, and IR, providing insights into the resin's synthesis and oil absorption mechanism. This analysis further substantiated the process by which oil molecules infiltrate the resin, resulting in its expansion.

(3) This study examines the impact of variations in MMA: SMA, MBA, PVA, and BPO ratios on the oil absorption capabilities of oil-absorbing resins to identify the composition that yields the highest oil absorption performance.

(4) The composite resin particles were utilized to plug fractures with widths ranging from 0.25 to 0.75 μm, demonstrating a filtration loss of 2.6 mL under high temperature and high pressure conditions in 0.75 μm cracks. Furthermore, the sealing mechanisms of the resin particles within geological formations were elucidated.

(5) The results of this study can be used in the field of drilling fluid plugging. The resin mentioned in this article can seal cracks of different widths, which can effectively solve the problem of difficult plugging in drilling operations.

## Data availability

The authors confirm that the data supporting the findings of this study are available within the article as its supplementary materials.

## Author contributions

Yang Bai: conceptualization, funding acquisition, writing – review & editing. Jianing He: investigation, data curation, writing – original draft. Jinsheng Sun: writing – review & editing, methodology. Ren Wang: supervision, formal analysis. Ruifang Wang: validation, project administration.

## Conflicts of interest

The authors declare no competing interests.
